# Technological arrangements for regulating access to healthcare: experience in the state of São Paulo

**DOI:** 10.11606/s1518-8787.2026060006604

**Published:** 2026-02-23

**Authors:** Mariana Prado Freire, Arthur Chioro, Luís Fernando Nogueira Tofani, André Luiz Bigal, Marília Cristina Prado Louvison

**Affiliations:** I Universidade de São Paulo. Faculdade de Saúde Pública. Departamento de Política, Gestão e Saúde. São Paulo, SP, Brasil; II Universidade Federal de São Paulo. Escola Paulista de Medicina. Departamento de Medicina Preventiva. São Paulo, SP, Brasil; III Faculdade de Medicina de Jundiaí. Departamento de Saúde Coletiva. Jundiaí, SP, Brasil

**Keywords:** Effective Access to Health Services, Health Care Coordination and Monitoring, Bed Occupancy, COVID-19

## Abstract

**OBJECTIVE:**

To describe and analyze the regulatory arrangements used to expand access to beds and health services during Covid-19.

**METHODS:**

This study, part of the Research for the SUS Program funded by Fundação de Amparo à Pesquisa do Estado de São Paulo (Fapesp) and the Secretaria de Ciência, Tecnologia, Inovação e Insumos Estratégicos em Saúde do Ministério da Saúde (Sctie-MS) Fapesp/Sctie used mixed methods to investigate the management of network care to face Covid-19 in São Paulo. Data was collected via an electronic questionnaire sent to managers in 645 municipalities between November 2021 and February 2022, with the support of the São Paulo Council of Municipal Health Secretaries (COSEMS-SP). The responses were analyzed quantitatively and qualitatively, with a focus on regulating access to healthcare.

**RESULTS:**

255 valid responses were collected (39.5% of São Paulo municipalities). Most municipalities regulated Covid-19 beds through the state regulation center (58%) and faced difficulties with access (53.7%), insufficient beds and 68.6% difficulties with hospitalization. The creation of new services was reported by 83.1%, and 70.0% implemented specific flows to expand access. In outpatient access, 54.5% used multiple regulation strategies. Financial resources were sufficient for 54.0% of the municipalities and 86.3% faced difficulties in acquiring supplies. Statistically significant associations were observed between municipal size and regulation variables.

**CONCLUSION:**

The Covid-19 pandemic has highlighted the importance of bed regulation and the unequal distribution between the public and private sectors. This study identified challenges such as insufficient beds, difficulties in admitting patients, and a reduction in outpatient services. Large municipalities have expanded beds through field hospitals and agreements with private hospitals. Small and medium-sized municipalities adopted regional strategies and multiple protocols. Regional coordination and state regulation were crucial in managing the response, but difficulties with supplies and human resources impacted on access. The incorporation of digital mechanisms and new regulatory strategies was important, despite the structural and political challenges that aggravated the emergency response.

## INTRODUCTION

The global spread of the SARS-CoV-2 virus, which began at the end of 2019, has resulted in a public health crisis on a global scale. Millions of people have been infected, and more than 7 million deaths have been recorded^1^, overloading health systems and making it necessary to re-evaluate conventional practices in the provision of health care. In addition to caring for the sick, implementing measures to contain the virus and maintaining essential social activities were major challenges. The scenario was aggravated by the difficulties encountered in the medical and hospital supply chain, logistical obstacles, human and financial resources, which exposed the fragility of health systems across the board^2,3^.

In this context, regulating access to healthcare during the pandemic has become a critical challenge. An analysis of the available literature shows that decision-makers, governments and health authorities began to use arrangements that could help manage limited resources. Various technological arrangements for regulating access have been identified^4^, with the aim of qualifying demand^5^, optimizing supply^4,6^, using technology to streamline processes^7,8^ and enabling patient care, with the implementation of field hospitals^9^, the adoption of differentiated protocols for access, and the use of digital health for patient care and monitoring being highlighted. In addition, the construction and strengthening of care networks^10^ played an important role in the response, identified in local contexts as well as regionally.

In Brazil and in other countries with universal health systems^11,12^, the regionalization of health systems through regions and care networks has been a strategy for increasing access, resizing supply and overcoming barriers to care^11^, often using technological regulation arrangements. Identifying the adoption of this type of strategy in the Unified Health System (SUS) reinforces the capillarity of health regulation actions, fostered by the National Regulation Policy. In addition, although the Brazilian regionalization process is still under construction^13^ and presents additional difficulties due to its size and scope^11^, its relevance and the expression of the results achieved^14^ are undeniable, including through governance mechanisms.

In this sense, the concept of governance is related to resource management, planning and policy formulation^14^. On a regional scale, governance mechanisms work to optimize installed capacity and rationalize care, generating economies of scale, integrating local systems, overcoming care barriers and access difficulties^14,15^. In Brazil, governance is advancing, producing spaces for articulation and solidarity between managers, but it faces challenges in terms of state protagonism and democratic legitimacy^10^, influenced by the allocation of resources in unequal logics.

The state of São Paulo, according to data from the Instituto Brasileiro de Geografia e Estatística (IBGE), concentrates around a third of the country’s gross domestic product, with strong economic activity in different areas, and is considered the most important economy in the nation. In health, it has the largest technological park, with the highest concentration of services and density of professionals, which coexist with significant challenges related to social inequalities^16^. For Lorenz et al.^17^, the evolution of the epidemic, starting in the capital and moving inland, coincides with the main state highways^17^ and the same with the epidemic peaks of 2020 and 2021. According to the authors, the profile of severe cases and deaths is similar to that found in other Brazilian countries and regions.

The São Paulo State Health Department coordinates and executes health actions through 17 regional health departments (DRS), which act as decentralized bodies. Coordination with the municipalities takes place mainly via the Regional Inter-Management Commission (CIR), a formal agreement body. The Council of Municipal Health Secretaries (COSEMS) plays a strategic role in aligning these actions. Despite weaknesses in regional coordination, the CIR has helped to expand access to healthcare, following established regionalization guidelines.

This article aims to describe and analyze the technological arrangements for regulating access to healthcare beds and services in the state of São Paulo during the Covid-19 pandemic. The importance of this study lies in the identification of productions and innovations used as facilitators to expand access to deal with the health crisis, understanding how these mechanisms were articulated in the SUS, how they contributed to overcoming the barriers imposed by the pandemic and their incorporation as a legacy.

## METHODS

This study is part of a research project under the Research Program for the SUS (PPSUS) funded by Fundação de Amparo à Pesquisa do Estado de São Paulo (Fapesp) and the Secretaria de Ciência, Tecnologia, Inovação e Insumos Estratégicos em Saúde do Ministério da Saúde (Sctie-MS) , using mixed methods with the aim of identifying the management of network care in the fight against Covid-19 in the state of São Paulo^18^. The data analyzed here was collected via an electronic questionnaire, answered by municipal health managers between November 2021 and February 2022. COSEMS-SP supported the dissemination of the survey and encouraging participation.

The questionnaire, with 39 questions (38 multiple choice and one discursive), was sent by e-mail to the 645 municipalities in São Paulo to be answered by the municipal manager/secretary or designate. The questions, prepared by the researchers and previously tested, were divided into four axes: Management, Health Surveillance, Network Assistance, and Vulnerabilities.

The multiple-choice answers were grouped and analyzed by the researchers in Excel tables, concentrating on the Management and Networked Care axis, with a focus on regulating access to healthcare. Answers to 18 questions were used, with descriptive analysis and the chi-square test (x^2^) to identify associations with the size of the municipalities, classified based on population as small, medium, large, and extra-large, with cut-off ranges established by the IBGE.

Cramer’s V was used as a measure of effect size, with the following cut-off points: > 0.1 - small; > 0.3 - medium; and > 0.5 - large^19^. The program used was the Statistical Package for the Social Sciences (SPSS) version 20^20^. The significance interval for positive associations was set at p < 0.05.

In addition, the answers to the discursive question were used to increase understanding of the quantitative findings, as pointed out by Creswell^21^. The narratives helped to identify technological regulation arrangements that act as facilitators for access to healthcare. AtlasTi 24 software was used, based on the elaboration and application of analysis category codes, which allowed the themes of interest to be brought together, as Friese^22^points out. The citations are identified using the references provided by the software, locating the document number, the coding number and the paragraph in which it is found (format: D:C¶P).

The research received funding from FAPESP and was approved by the Research Ethics Committee of the Universidade Federal de São Paulo, under CAAE 45679521.6.0000.5505. The participants were given a free and informed consent form at the beginning of the electronic questionnaire and before the interviews were carried out, they agreed to take part in the study, and the respondents were guaranteed confidentiality and anonymity.

## RESULTS

In total, 340 responses were collected, excluding questionnaires answered in duplicate, leaving 255 valid responses (39.5% of São Paulo municipalities), representing all the health regions in the state of São Paulo, delimited by the seventeen DRS. Two-thirds were provided by the managers of the Municipal Health Departments (SMS), and most of the participating municipalities are small (51.4%), as shown in Table 1.

**Table 1:** Characterization of the sample.

Responding municipalities		n	%
		255	39.5
Responding municipalities by DRS	DRS 15 São José Rio Preto	37	14.51
	DRS 6 Bauru	35	13.73
	DRS 9 Marilia	28	10.98
	DRS 1 Greater São Paulo	20	7.84
	DRS 11 Presidente Prudente	15	5.88
	DRS 17 Taubaté	15	5.88
	DRS 13 Ribeirão Preto	14	5.49
	DRS 16 Sorocaba	13	5.10
	DRS 2 Araçatuba	13	5.10
	DRS 7 Campinas	12	4.71
	DRS 10 Piracicaba	9	3.53
	DRS 3 Araraquara	9	3.53
	DRS 5 Barretos	8	3.14
	DRS 8 Franca	8	3.14
	DRS 12 Registro	7	2.75
	DRS 4 Baixada Santista	7	2.75
	DRS 14 São João Boa Vista	5	1.96
Respondent	Municipal manager	171	67
	Representative appointed by the municipal manager	84	33
Size of municipality	Small (up to 20,000 inhabitants)	131	51.37
	Medium (20,001 to 100,000 inhabitants)	83	32.55
	Large (100,001 to 500,000 inhabitants)	35	13.73
	Extra-large (over 500,000 inhabitants)	6	2.35

DRS: regional health departments.

The results were grouped between hospital/urgency regulation (Table 2) and outpatient/non-Covid regulation and other aspects (Table 3), using data from the Management and Network Assistance axes. The findings of association with the size of the municipalities are described below and shown in Table 4.

**Table 2:** Hospital and emergency regulation.

			n	%
Regulation of Covid-19 beds through a central regulation center	Management axis	State / CROSS	148	58.0
		Regulated by more than one center	76	29.8
		Regional	11	4.3
		Not regulated by central regulators	10	3.9
		Municipal	7	2.7
		Don’t know	3	1.2
Difficulty admitting patients	Network Assistance	Had difficulties with both	175	70.3
		Difficulties with surgeries and non-Covid-19 cases	32	12.9
		No difficulties	23	9.2
		Had difficulties with hospitalization of Covid-19 cases in ward or ICU	19	7.6
Regulation of Covid beds - difficulties in accessing beds	Management	Insufficient ward, ventilator, or ICU beds	137	53 .7
		Insufficient beds associated with other factors	60	23.5
		No difficulties encountered in accessing Covid-19 beds	38	14.9
		Don’t know	11	4.3
		Problems other than insufficient beds	9	3.5
Situations faced by the private sector	Axis Ges so	Various situations faced with the private sector - beds and other resources	107	42.0
		None	77	30.2
		Situations faced with the private sector not involving hospitalization beds	56	22.0
		Situations with the private sector exclusively involving inpatient beds	15	5.9
Covid bed regulation - protocols used	Management axis	More than one protocol	109	42.7
		State protocol	91	35.7
		Regional protocol	28	11.0
		Municipal protocol	19	7.5
		Can’t answer	4	1.6
		Did not use protocols	2	0.8
		National protocol	2	0.8
Creation of new services in the care network	Network Assistance	Yes	212	83.1
		No	39	15.2
Strategies adopted in urgent and emergency services	Networked Care	Specific areas/flows for Covid care in existing services	179	70.2
		Services created or adapted for Covid care	131	51.3
		Specific emergency services set up for Covid care	43	16.8
		Municipality does not have an emergency service	29	11.3
		There has been no change in the emergency network	25	9.8
		Specific Samu units have been assigned to Covid-19 care	17	6.6
		Telehealth for emergency services implemented	14	5.4
		Don’t know	3	1.1

CROSS: São Paulo State Health Services and Supply Regulation Center; ICU: intensive care unit; SAMU: Mobile Emergency Care Service.

**Table 3:** Outpatient regulation / non-Covid and other factors.

			n	%
Regulation strategies adopted	Management axis	Multiple strategies	139	54.5
		Other outpatient regulation strategies	39	15.3
		Qualification / monitoring of waiting lines,	28	11.0
		No action was taken,	18	7.1
		Implementation of risk classification protocols	14	5.5
		Don’t know	12	4.7
		Teleregulation	3	1.2
		Matrix Support	2	0.8
Change in the supply of specialized consultations	Network Assistance	Decreased	187	73.3
		Increased	36	14.1
		Remained stable	29	11.4
		Don’t know	3	1.2
Change in the supply of diagnostic support tests	Network Assistance	Decreased	163	63.9
		Increased	58	22.7
		Remained stable	31	12.2
		Don’t know	3	1.2
Change in the offer of therapeutic procedures	Network Assistance	Decreased	172	67.5
		Increased	47	18.4
		Remained stable	28	11.0
		Don’t know	8	3.1
Change in the supply of surgeries	Network Assistance	Decreased	210	82.4
		Increased	3 1	12.2
		Don’t know	8	3.1
		Remained stable	6	2.4
Change in teleservice offer	Network Assistance	Increased	93	36.5
		Don’t know	72	28.2
		Decreased	54	21.2
		Remained stable	36	14.1
Were the financial resources sufficient?	Management axis	Yes	138	54.1
		No	100	39.2
		Don’t know	17	6.7
Changes in the workforce - emergency services and SAMU	Management	There was an increase in staff	116	45.5
		No change	80	31.4
		Don’t know how to answer	30	11.8
		There has been a reduction in staff, including departures and resignations	29	11.4
Changes in the workforce - hospital	Management	There was an increase in staff	125	49.0
		No change	57	22.4
		Don’t know how to answer	47	18.4
		There has been a reduction in staff, including departures and resignations	26	10.2
Changes in the workforce - specialized care	Management	No change	134	52.5
		There was a reduction in staff, including leaves of absence and resignations	45	17.6
		There was an increase in staff	40	15.7
		Can’t answer	34	13.3
		There has been a reduction in staff	2	0.8
Difficulties in acquiring materials/inputs	Management	Multiple difficulties	220	86.3
		Lack of products on the market	14	5.5
		No difficulties	8	3.1
		Abusive prices	6	2.4
		Administrative / legal obstacles to purchase	4	1.6
		Insufficient municipal finances / budget	2	0.8
		Don’t know how to answer	1	0.4

SAMU: Mobile Emergency Care Service.

**Table 4:** Association with size.

Variables		Size				Cramer value	p-value
		Extra large	Large	Medium	Small		
Were the financial resources sufficient?	No	4	25^a^	44^a^	27^a^	0.309	< 0.001
	Don’t know	0	2	8	7		
	Yes	2	8^a^	31^a^	97^a^		
Is regulation of Covid-19 beds done through a regulation center?	State/CROSS	1^a^	15	52	80	0.315	< 0.001
	Municipal	3^a^	4^a^	0	0^a^		
	Not regulated by central regulators	0	1	3	6		
	Don’t know	0	0	0	3		
	Regional	0	0	4	7		
	Regulated by more than one central office	2	15	24	35		
Covid bed regulation - protocols used	More than one protocol	1	20	34	54	0.312	< 0.001
	Don’t know how to answer	0	1	0	3		
	Did not use protocols	0	0	1	1		
	State protocol	0	7^a^	36	48		
	Municipal protocol	5^a^	6^a^	5	3^a^		
	National protocol	0	0	1	1		
	Regional protocol	0	1	6	21^a^		
Covid bed regulation - difficulties in accessing beds	Insufficient beds associated with other factors	0	12	26^a^	22^a^	0.241	< 0.001
	Insufficient ward beds, ventilator support or ICU beds	1	14	48	74		
	No difficulties encountered in accessing Covid-19 beds	3^a^	2	6^a^	27^a^		
	Don’t know	1	2	3	5		
	Problems other than insufficient beds	1	5^a^	0^a^	3		
Outpatient regulation - non-Covid access	Implementation of risk classification protocols	2^a^	1	7	4	0.191	0.146
	Matrix Support	0	0	1	1		
	Multiple strategies	4	25^a^	42	68		
	No action was taken.	0	1	2^a^	15^a^		
	Don’t know	0	1	5	6		
	Other outpatient regulation strategies	0	3	13	23		
	Qualification / monitoring of waiting lines	0	4	12	12		
	Tele-regulation	0	0	1	2		
Situations faced with the private sector	None	0	1^a^	26	50^a^	0.224	< 0.001
	Situations faced with the private sector not involving hospitalization beds	0	6	12^a^	38^a^		
	Situations with the private sector - involving inpatient beds	1	3	5	6		
	Situations with the private sector involving beds among others	5^a^	25^a^	40	37^a^		
Difficulty admitting patients	Don’t know	1	1	1	3	0.180	0.017
	No difficulty	0	2	3^a^	18^a^		
	Had difficulty with both	3	24	69^a^	79^a^		
	Had difficulties with surgeries and non-Covid-19 cases	2	6	7	17		
	Had difficulties with hospitalization of Covid-19 cases in ward or ICU	0	2	3	14^a^		
Changes in the workforce - emergency services and Samu	There has been an increase in staff	5	22^a^	45^a^	44^a^	0.195	0.001
	There has been a reduction in staff, including departures and refusals	0	7	10	12		
	No change	1	4^a^	23	52^a^		
	Don’t know how to answer	0	2	5^a^	23^a^		
Changes in the workforce - hospital	There was an increase in staff	5	25^a^	53^a^	42^a^	0.259	0.000
	There was a reduction in staff, including departures and resignations	0	6	10	10		
	No change	0	1^a^	7^a^	49^a^		
	Don’t know how to answer	1	3	13	30^a^		
Changes in the workforce - specialized care	There was an increase in staff	1	3	20^a^	16	0.194	0.004
	There was a reduction in staff	0	1	1	0		
	There was a reduction in staff, including leaves of absence and resignations	1	13^a^	15	16^a^		
	No change	3	17	41	73		
	Don’t know how to answer	1	1	6	26^a^		
Difficulties in acquiring materials/inputs	Administrative/legal obstacles to acquisition	0	0	2	2	0.203	0.026
	Lack of products on the market	1	0	3	10		
	Insufficient municipal finances/budget	0	1	0	1		
	multiple difficulties	4	33	78^a^	105^a^		
	No difficulties	0	1^a^	0^a^	7^a^		
	abusive prices	0	0	0	6		
Change in the supply of specialized consultations	Increased	0	4	15	17	0.076	0.882
	Decreased	5	26	59	97		
	Remained stable	1	4	9	15		
	Don’t know	0	1	0	2		
Change in the offer of diagnostic support tests	Increased	1	5	27^a^	25	0.171	0.008
	Decreased	4	25	49	85		
	Remained stable	0	5	7	19		
	Don’t know	1^a^	0	0	2		
Change in the offer of therapeutic procedures	Increased	1	4	19	23	0.073	0.903
	Decreased	5	25	53	89		
	Remained stable	0	4	9	15		
	Don’t know	0	2	2	4		
Change in the supply of surgeries	Increased	1	3	13	14	0.108	0.443
	Decreased	4	30	68	108		
	Remained stable	0	1	2	3		
	Don’t know	1	1	0	6		
Change in customer service	Increased	5^a^	18^a^	28	42	0.140	0.094
	Decreased	0	4	16	34		
	Remained stable	1	5	10	20		
	Don’t know	0	8	29	35		

SAMU: Mobile Emergency Care Service; ICU: intensive care unit.

^a^Data statistically significant by analysis of adjusted standardized residuals.

### Hospital/Urgency Regulation

Most municipalities (58.0%) regulated beds exclusively for Covid-19 through the São Paulo State Health Services and Supply Regulation Center (CROSS). However, there were difficulties with access: 53.7% reported insufficient beds and 70.3% faced problems admitting patients. As for hospitalization protocols, 42.7% used more than one protocol, as shown in Table 2.

The creation of new services for suspected cases of Covid-19, such as care centers to deal with Covid-19, was reported by 83.1% of respondents. In emergency services, 70.2% have implemented specific flows and 51.3% have created or adapted services to deal with Covid-19. The relationship between the public and private sectors was expanded, including the expansion of the number of beds (42.0%). On the other hand, 21.5% of public managers received requests for transfer from the private sector to public beds.

### Outpatient/Non-Covid Regulation

In terms of access to outpatient healthcare not related to Covid-19, 54.5% of respondents used multiple regulation strategies, such as monitoring queues, risk classification protocols, matrix support, and teleregulation. There was a reduction in specialized consultations, therapeutic procedures, diagnostic tests and surgeries, as shown in Table 3.

### Other Factors Associated with Regulation

Other factors have interfered with access to healthcare during the pandemic, such as the availability of financial resources, which 54.0% of municipalities considered sufficient, while 39.0% disagreed. There was an increase in the number of staff in the emergency services and the Mobile Emergency Care Service (SAMU) (45.5%) and in the hospital sector (49.0%), but in specialized care 52.5% reported no change and 17.6% recorded a reduction due to sick leave. In addition, 86.3% faced difficulties in acquiring materials and supplies.

### Associations with the Size of Municipalities

Table 4 shows the association between the size of the municipality and the variables of interest. Regarding hospital beds, the majority of small municipalities were regulated by a state/CROSS center (p < 0.001). Statistical significance was also found in the use of Covid-19 bed regulation protocols (p < 0.001). Small municipalities were more likely to adopt multiple protocols, while large municipalities more often adopted the state protocol.

As for strategies to ensure access to outpatient services not related to Covid-19, there was a significant association between large municipalities and the use of multiple strategies (p < 0.05), such as outpatient regulation, queue qualification, risk protocols, matrix support and teleregulation. Small municipalities, on the other hand, were associated with the absence of actions (p < 0.05). Small and medium-sized municipalities also adopted various strategies, but without statistical significance (p = 0.146).

The associations were less obvious for variables related to the provision of outpatient services, such as specialized consultations and surgeries. There was statistical significance in the reduction of diagnostic tests (p = 0.008), but not in specialized consultations. Sufficiency of financial resources was associated with the size of the municipality (p < 0.001), with small municipalities reporting greater sufficiency. Managers in large and medium-sized municipalities, on the other hand, reported insufficiency.

There was also a significant association between municipal size and an increase in staff in emergency services and SAMU (p = 0.001) and in hospital care (p = 0.000). Small municipalities had more “unchanged” workforces and faced more difficulties in acquiring supplies, especially due to a lack of products on the market (p = 0.026).

The results presented were categorized into barriers and facilitators of access, highlighting the technological regulation arrangements used, helping to analyze the importance of these mechanisms. At this stage of the study, narratives from managers were highlighted which contribute to understanding the dynamics of the use of arrangements in facilitating and tackling the barriers encountered in the health emergency (Figure).

**Figure:**
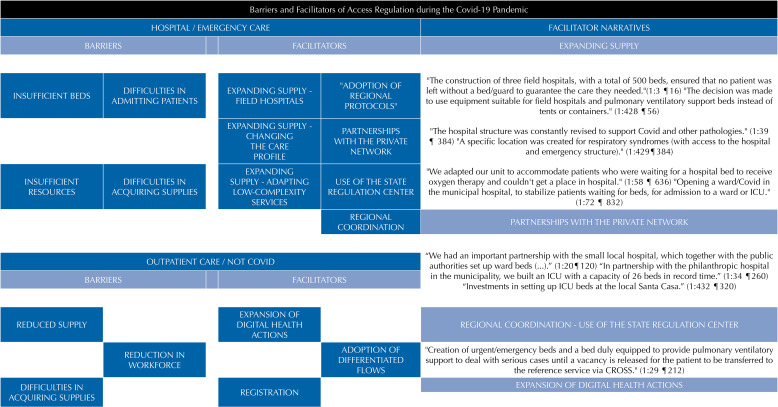
Barriers and facilitators of regulation during the pandemic.

Note: The identification of citations uses the references provided by the AtlasTi software in the format D:C ¶ P D=document number, C=code number and P=paragraph number. ICU: intensive care unit; CROSS: São Paulo State Health Services and Supply Regulation Center.

## DISCUSSION

From a broader perspective of access to healthcare, based on the elements that make up the regulation process, the findings described in this study reinforce barriers such as insufficient beds, difficulties in admitting patients and a reduction in the supply of outpatient services, in line with the current literature^23,24^ on coping with the pandemic. However, facilitators such as the relationship between the public and private sectors and regional coordination were revealed. Regarding the distribution of resources, the asymmetry identified has had different effects in different regions of the state.

The Covid-19 pandemic has highlighted the importance of financial resources in the management and organization of health systems. In Brazil, the SUS is funded on a tripartite basis, but the unequal distribution has created significant challenges^25^. It is important to note that municipal elections took place in Brazil during the first year of the pandemic, bringing other elements into the political scenario and the allocation of resources^26^. The larger municipalities, with greater responsibility for managing the crisis, faced insufficient resources to expand hospital structures and technologically expand their health units. On the other hand, the small municipalities, which make up the majority, reported that resources were sufficient, although they had low technological density. Complexity and bureaucracy also imposed difficulties in the use of resources, aggravating the problems in dealing with the pandemic.

The seriousness of the infectious situation caused by the SARS-Cov2 virus indicates the centrality of hospital beds in building the emergency response. According to a report by the Ministry of Health’s Extraordinary Secretariat for Coping with Covid, 14% of patients with Covid-19 have progressed to serious conditions in Brazil^27^, requiring oxygen therapy and intensive care units. Considering this, there was a 23.5% increase in the number of intensive care unit (ICU) beds in Brazil in the first six months of the pandemic^28^. However, although the data is surprising, this expansion has maintained the profile of unequal distribution of beds, in regional terms and in the division between public and private beds, accentuating the structural flaw in the distribution of ICU beds in Brazil^29^.

The state of São Paulo, which has one of the largest number of ICU beds in the country^30^, became the epicenter of the pandemic, especially its capital^31^, and was the first Brazilian state to be hit. The inequalities in the distribution of beds in São Paulo are strongly linked to the discrepancy between the number of public and private beds, considering the dependent population. The lack of beds was one of the main barriers identified and mobilized the use of regulatory arrangements in an attempt to minimize the impacts caused.

The organization of the response through the expansion of beds is portrayed in the data collected, where it is also possible to identify the arrangements that were used to enable the expansion of the care network, acting as facilitators of access. In large and extra-large municipalities, the increase in beds was largely the result of expanding the existing structure, setting up field hospitals^32^ and contracting with private hospitals.

The larger municipalities, while on the one hand benefiting from the fact that they already had a hospital and emergency network in place, on the other hand honored regional agreements, serving as a reference for small municipalities with insufficient or even non-existent hospital and emergency networks^33^.

Medium-sized municipalities, on the other hand, used arrangements such as changing the care profile of certain wards, or even the hospital as a whole, as a way of increasing access to beds. In small municipalities, other arrangements have been implemented to guarantee initial emergency care, until transfer to larger centers, respecting the regional logic of care and the agreements made in the different health regions^33^. In this context, the managers of small municipalities highlighted the measures adopted to minimize the difficulties of hospitalization, creating temporary or transitional structures until access to adequate resources was possible.

The organization of care in the state of São Paulo reinforces the importance of regional coordination between municipalities in the composition of a comprehensive regional care network, historically established through agreements between managers^11^. In the context of emergency care, the commitment to the constitution of regions and networks adopted in São Paulo served as a facilitator for the organization of regional reference grids, covering different levels of complexity, challenges for regional regulation, distribution of resources and patient transport.

As a way of operationalizing the flows/grids established for care, the central regulation offices have emerged as a central cog in organizing the response. The predominance of the state regulation center and the adoption of multiple protocols for regulating access to beds corroborate the notion that the CIRs are powerful spaces for developing new care arrangements to overcome access difficulties^34^ and strengthen the commitment to the creation of regions and networks as a way of moving forward in the construction of a health system that addresses the problems of access and adequacy of supply^11^.

Through municipal protagonism, agreements built in the territories were put into practice, the result of shared planning and management efforts^14^, forged in a scenario of intense political mobilization, tensioned by private interests, the media, and the judiciary. CROSS acted as an instrument of regional governance in the regulation of beds, serving as a link between entities, in an attempt to minimize the historical weakening of its role in the spheres of agreement^13^, the result of the widening participation of service providers in management spaces, the distancing of financial resources and the reduced negotiating power of the regions. This scenario was aggravated by the absence of the federal government in coordinating the response to the pandemic, relegating responsibility for building the response to the sub-national entities.

On the other hand, in the state of São Paulo alone, the private sector had more than 50% of the existing ICU beds, according to data from before the pandemic^29^. The health emergency intensified existing relations between the public and private sectors and provided an opportunity to establish new cooperation formats. The medium-sized municipalities that established technological regulation arrangements to guarantee patients’ access to specialized beds relied heavily on the support of the private sector, including philanthropic hospitals, establishing partnerships or expanding existing agreements to make Covid-19 beds available.

However, the private sector has also resorted to the supply provided by SUS, evidence of the collapse also faced by private hospitals, health plans and insurance^29,35^. Although the implementation of a single queue for access to Covid beds has been discussed in Brazil, unifying public and private supply, as well as the possibility of decrees for the requisition of beds by the public authorities, the proposal has not advanced^35^.

The expansion of the workforce, which is crucial to increasing access, also represented a major challenge for managers, both because of the scarcity of human resources available and because of the inexperience and need to train the contingent of workers in a short space of time. The high rate of contamination among professionals, especially those identified as the “front line”, limited actions, affecting supply and consequently access to health services^36^. Protecting professionals from the increased risk of infection required efforts to provide personal protective equipment and adequate training for handling personal protective equipment and patients^37^.

Moreover, the difficulty in acquiring supplies is also an important obstacle faced, along with the difficulty in accessing beds. Although strategies to deal with the issue of supplies have been identified, limitations in the adequate supply of hospitals have led to reports of a severe operational challenge faced by hospital managers, motivated mainly by the scarcity of products, but also by the practice of exorbitant prices, which has further restricted access^38^.

Changing the supply of services in specialized care was also pointed out as a challenge. Reducing the number of elective surgeries and specialized consultations was seen as a solution^36,41^ to increase the supply of care, reduce the exposure of users and workers and optimize resources, but it also caused problems due to the reduction in supply, making access to historically sensitive and problematic health services even more difficult^42^. Concern about chronic diseases considers the impact of Covid-19 and the need to re-establish links with patients and outpatient treatment. The reduction in outpatient supply has had disastrous effects on specialized health care, increasing the ongoing challenge at this level of care.

The incorporation of digital health mechanisms as part of the strategy to expand access has strengthened and innovated through the creation of telemonitoring strategies, multi-professional care, mental health care and clinical management of hospitalized patients. Important tools for health education were used intensively, such as video resources, meetings between managers via digital platforms, the use of messaging applications (WhatsApp)^43^ to assist in the bed regulation process, among others, even though they had already been used and recognized in other contexts.

The data from this study reveals the complexity and challenges faced by the SUS during the Covid-19 pandemic. The insufficiency of beds and the inequality in their distribution, highlighting inequalities between the public and private sectors, were central problems that hampered the response to the emergency. Municipalities have led efforts to increase the supply of care, expanding and readjusting the care network. Technological arrangements for regulating access to healthcare, such as access protocols and changes in the care profile, were crucial in tackling complex problems. Regional coordination and the use of regulation centers were key to mitigating difficulties, reinforcing the importance of shared and well-structured governance.

However, efforts to increase access to beds sacrificed outpatient services, reducing the supply and the outpatient workforce and concentrating resources on hospital care. Technological arrangements, such as matrix support, queue qualification and the use of digital tools and telemedicine, have helped to guarantee access. Studies on post-pandemic access to healthcare will be essential to understand the impact of these arrangements and guide future responses to health emergencies.

The use of an electronic questionnaire is a limitation of the study, which may have excluded participants with less digital familiarity and made it difficult to capture complex realities.

Strengthening spaces for regional agreement, which operate through robust governance mechanisms, could be important for future responses, ensuring networked assistance mechanisms and funding for actions. The expansion of the workforce and the adequate supply of essential materials can also contribute, minimizing the effects on specialized outpatient health actions.
