# Supplementary material for: Technological arrangements for regulating access to healthcare: experience in the state of São Paulo
Source: Rev Saude Publica. 2026 Feb 23;60:e5. doi: 10.11606/s1518-8787.2026060006604 (PMC12928190; doi:10.11606/s1518-8787.2026060006604)
Supplement: Translation

# Translation: Arranjos tecnológicos de regulação do acesso à saúde: experiência do estado de São Paulo

**Authors:** Mariana Prado Freire, Arthur Chioro, Luís Fernando Nogueira Tofani, André Luiz Bigal, Marília Cristina Prado Louvison

**Affiliations:** I Universidade de São Paulo. Faculdade de Saúde Pública. Departamento de Política, Gestão e Saúde. São Paulo, SP, Brasil; II Universidade Federal de São Paulo. Escola Paulista de Medicina. Departamento de Medicina Preventiva. São Paulo, SP, Brasil; III Faculdade de Medicina de Jundiaí. Departamento de Saúde Coletiva. Jundiaí, SP, Brasil

**Keywords:** Acesso Efetivo aos Serviços de Saúde, Regulação e Fiscalização em Saúde, Ocupação de Leitos, COVID-19

## Abstract

**OBJETIVO:**

Descrever e analisar os arranjos de regulação empregados para a ampliação do acesso a leitos e serviços de saúde durante a covid-19.

**MÉTODOS:**

Estudo parte de pesquisa Programa Pesquisa para o SUS financiada pela Fundação de Amparo à Pesquisa do Estado de São Paulo (Fapesp) e da Secretaria de Ciência, Tecnologia, Inovação e Insumos Estratégicos em Saúde do Ministério da Saúde (Sctie-MS), utilizando métodos mistos, investigou a gestão do cuidado em rede para enfrentar a covid-19 em São Paulo. Dados foram coletados via questionário eletrônico, enviado a gestores de 645 municípios entre novembro de 2021 e fevereiro de 2022, com apoio do Conselho dos Secretários Municipais de Saúde de São Paulo (Cosems-SP). As respostas foram analisadas quantitativa e qualitativamente, com foco na regulação do acesso à saúde.

**RESULTADOS:**

Foram coletadas 255 respostas válidas (39,5% dos municípios paulistas). A maioria dos municípios regulou leitos covid-19 pela central de regulação estadual (58%) e enfrentou dificuldades de acesso (53,7%), insuficiência de leitos e 68,6% dificuldades para internação. A criação de novos serviços foi relatada por 83,1%, e 70,0% implementaram fluxos específicos para ampliar o acesso. No acesso ambulatorial, 54,5% usaram múltiplas estratégias de regulação. Recursos financeiros foram suficientes para 54,0% dos municípios e 86,3% enfrentaram dificuldades na aquisição de insumos. Associações estatísticas significativas foram observadas entre porte municipal e variáveis de regulação.

**CONCLUSÃO:**

A pandemia de covid-19 destacou a importância da regulação de leitos e a desigualdade na distribuição entre os setores público e privado. Este estudo identificou desafios como a insuficiência de leitos, dificuldades na internação de pacientes e a redução de serviços ambulatoriais. Municípios de grande porte ampliaram leitos por meio de hospitais de campanha e acordos com hospitais privados. Já os pequenos e médios adotaram estratégias regionais e protocolos múltiplos. A articulação regional e a regulação estadual foram cruciais na gestão da resposta, mas as dificuldades com insumos e recursos humanos impactaram o acesso. A incorporação de mecanismos digitais e novas estratégias de regulação foram importantes, apesar dos desafios estruturais e políticos que agravaram a resposta emergencial.

## INTRODUÇÃO

A disseminação global do vírus SARS-CoV-2, com início no final de 2019, resultou em uma crise de saúde pública em escala global. Milhões de pessoas foram infectadas e foram contabilizadas mais de 7 milhões de mortes^1^, sobrecarregando os sistemas de saúde, o que tornou necessária a reavaliação das práticas convencionais na prestação de assistência à saúde. Além do cuidado com os doentes, a implementação de medidas para conter o vírus e a manutenção de atividades sociais essenciais foram importantes desafios. O cenário foi agravado pelas dificuldades encontradas na cadeia de suprimentos médicos e hospitalares, obstáculos logísticos, de recursos humanos e financeiros, que expuseram a fragilidade dos sistemas de saúde indistintamente^2,3^.

Neste contexto, a regulação do acesso à saúde durante a pandemia tornou-se um desafio crítico. Analisando a literatura disponível é possível verificar que tomadores de decisão, governantes e autoridades de saúde passaram a utilizar arranjos que pudessem auxiliar na gestão dos recursos limitados. Foram identificados diversos arranjos tecnológicos de regulação do acesso^4^, com o objetivo de qualificar a demanda^5^, otimizar a oferta^4,6^, empregar tecnologia para agilizar processos^7,8^ e viabilizar a atenção aos pacientes, com destaque para a implementação de hospitais de campanha^9^, adoção de protocolos diferenciados para o acesso e o uso de saúde digital para o atendimento e monitoramento de pacientes. Além disso, a construção e fortalecimento de redes de atenção^10^desempenharam importante papel na resposta, identificada em contextos locais, bem como de forma regionalizada.

No Brasil e em outros países com sistemas universais de saúde^11,12^, a regionalização dos sistemas de saúde por meio de regiões e redes de cuidado tem sido estratégia para ampliar o acesso, redimensionar a oferta e superar entraves assistenciais^11^, frequentemente empregando arranjos tecnológicos de regulação. A identificação da adoção deste tipo de estratégias no Sistema Único de Saúde (SUS) reforça a capilaridade das ações de regulação em saúde, fomentadas pela Política Nacional de Regulação. Adicionalmente, ainda que o processo de regionalização brasileiro se encontre em construção^13^ e apresente dificuldades adicionais em virtude de sua dimensão e abrangência^11^, é inegável a sua relevância e a expressão dos resultados conquistados^14^, inclusive, por meio de mecanismos de governança.

Neste sentido, o conceito de governança está relacionado à gestão de recursos, ao planejamento e à formulação de políticas^14^. Em escala regional, os mecanismos de governança atuam na otimização da capacidade instalada e racionalização da assistência, gerando economia de escala, integração de sistemas locais, superação de barreiras assistenciais e dificuldades no acesso^14,15^. No Brasil, a governança avança produzindo espaços de articulação e solidariedade entre gestores, mas enfrenta desafios quanto ao protagonismo estadual e legitimidade democrática^10^, influenciados pela alocação de recursos em lógicas desiguais.

O estado de São Paulo, segundo dados do Instituto Brasileiro de Geografia e Estatística (IBGE), concentra cerca de um terço do produto interno bruto nacional, com forte atividade econômica em diferentes áreas, e é considerada a economia mais importante da nação. Na saúde, reúne o maior parque tecnológico, com a maior concentração de serviços e densidade de profissionais, que coexistem com importantes desafios relacionados às desigualdades sociais^16^. Para Lorenz et al.^17^, a evolução da epidemia, com início na capital e avanço para o interior, coincide com as principais rodovias estaduais^17^e o mesmo com os picos epidêmicos de 2020 e 2021. Segundo os autores, o perfil dos casos graves e óbitos se assemelha ao encontrado em outros países e regiões brasileiras.

A Secretaria Estadual de Saúde de São Paulo coordena e executa ações de saúde por meio de 17 departamentos regionais de saúde (DRS), que atuam como instâncias descentralizadas. A articulação com os municípios ocorre, principalmente, via Comissão Intergestores Regional (CIR), instância formal de pactuação. O Conselho dos Secretários Municipais de Saúde (Cosems) tem papel estratégico no alinhamento dessas ações. Apesar de fragilidades na articulação regional, a CIR favoreceu a ampliação do acesso à saúde, seguindo diretrizes de regionalização já estabelecidas.

O presente artigo tem como objetivo descrever e analisar os arranjos tecnológicos de regulação do acesso a leitos e serviços de saúde no estado de São Paulo durante a pandemia de covid-19. A importância deste estudo reside na identificação de produções e inovações utilizadas como facilitadores de ampliação do acesso para o enfrentamento da crise sanitária, compreendendo de que forma esses mecanismos se articularam no SUS, como contribuíram na superação das barreiras impostas pela pandemia e sua incorporação como legado.

## MÉTODOS

Este estudo, parte de uma pesquisa do Programa de Pesquisa para o SUS (PPSUS) financiada pela Fundação de Amparo à Pesquisa do Estado de São Paulo (Fapesp) e da Secretaria de Ciência, Tecnologia, Inovação e Insumos Estratégicos em Saúde do Ministério da Saúde (Sctie-MS) Fapesp/Sctie-MS, utilizando métodos mistos com o objetivo de identificar a gestão do cuidado em rede no enfrentamento da covid-19 no estado de São Paulo^18^. Os dados aqui analisados foram coletados via questionário eletrônico, respondido por gestores municipais de saúde entre novembro de 2021 e fevereiro de 2022. A divulgação da pesquisa e estímulo à participação teve o apoio do Cosems-SP.

O questionário, com 39 perguntas (38 de múltipla escolha e uma discursiva), foi enviado por e-mail aos 645 municípios paulistas para ser respondido pelo gestor/secretário municipal ou designado. As questões, elaboradas pelos pesquisadores e testadas previamente, foram divididas em quatro eixos: Gestão, Vigilância em Saúde, Assistência em Rede e Vulnerabilidades.

As respostas de múltipla escolha foram agrupadas e analisadas pelos pesquisadores em tabelas do Excel, concentradas no eixo Gestão e Assistência em Rede, com foco na regulação do acesso à saúde. Foram utilizadas respostas de 18 questões, com análise descritiva e teste de qui-quadrado (x^2^) para identificar associações com o porte dos municípios, classificados com base populacional como pequeno, médio, grande e extragrande, com faixas de corte estabelecidas pelo IBGE.

Como medida de tamanho de efeito foi adotado o V de Cramer, seguindo os seguintes pontos de corte: > 0,1 – pequeno; > 0,3 – médio; e > 0,5 – grande^19^. O programa utilizado foi o *Statistical Package for the Social Sciences* (SPSS) versão 20^20^. O intervalo de significância para associações positivas foi arbitrado em p < 0,05.

Além disso, foram utilizadas as respostas da questão discursiva, com o objetivo de aumentar a compreensão dos achados quantitativos, como aponta Creswell^21^. As narrativas auxiliaram na identificação de arranjos tecnológicos de regulação atuando como facilitadores para o acesso à saúde. Foi utilizado o *software* AtlasTi 24, a partir da elaboração e aplicação de códigos de categorias de análise, que permitiram a reunião dos temas de interesse, como aponta Friese^22^. As citações estão identificadas utilizando as referências fornecidas pelo software, localizando número do documento, número da codificação e o parágrafo em que se encontra (formato: D:C¶P)

A pesquisa recebeu financiamento da Fapesp e foi aprovada pelo Comitê de Ética em Pesquisa da Universidade Federal de São Paulo, sob CAAE 45679521.6.0000.5505. Os participantes receberam o termo de consentimento livre e esclarecido, apresentado no início do questionário eletrônico e antes da realização das entrevistas, concordaram em participar do estudo e foi garantido sigilo e anonimato aos respondentes.

## RESULTADOS

No total, 340 respostas foram coletadas, sendo excluídos os questionários respondidos em duplicidade, restando 255 respostas válidas (39,5% municípios paulistas), representando todas as regiões de saúde do Estado de São Paulo, delimitados pelos dezessete DRS. Dois terços foram fornecidos pelos gestores titulares das Secretarias Municipais de Saúde (SMS) e a maioria dos municípios participantes são de pequeno porte (51,4%), conforme apresentado na Tabela 1.

**Tabela 1:** Caracterização da amostra.

Municípios respondentes		n	%
		255	39,5
Municípios respondentes por DRS	DRS 15 São José Rio Preto	37	14,51
	DRS 6 Bauru	35	13,73
	DRS 9 Marilia	28	10,98
	DRS 1 Grande São Paulo	20	7,84
	DRS 11 Presidente Prudente	15	5,88
	DRS 17 Taubaté	15	5,88
	DRS 13 Ribeirão Preto	14	5,49
	DRS 16 Sorocaba	13	5,10
	DRS 2 Araçatuba	13	5,10
	DRS 7 Campinas	12	4,71
	DRS 10 Piracicaba	9	3,53
	DRS 3 Araraquara	9	3,53
	DRS 5 Barretos	8	3,14
	DRS 8 Franca	8	3,14
	DRS 12 Registro	7	2,75
	DRS 4 Baixada Santista	7	2,75
	DRS 14 São João Boa Vista	5	1,96
Respondente	Gestor municipal	171	67
	Representante indicado pelo gestor municipal	84	33
Porte do município	Pequeno (até 20,000 habitantes)	131	51,37
	Médio (20,001 a 100,000 habitantes)	83	32,55
	Grande (100,001 a 500,000 habitantes)	35	13,73
	Extragrande (acima de 500 mil habitantes)	6	2,35

DRS: departamentos regionais de saúde.

Os resultados foram agrupados entre a regulação hospitalar/urgência (Tabela 2) e a regulação ambulatorial/não covid e outros aspectos (Tabela 3), utilizando os dados dos eixos Gestão e Assistência em Rede. Os achados de associação com o porte dos municípios estão descritos a seguir e representados na Tabela 4.

**Tabela 2:** Regulação hospitalar e de urgência.

			n	%
Regulação de leitos covid feita através de central de regulação	Eixo Gestão	Estadual / Cross	148	58,0
		Regulados por mais de uma central	76	29,8
		Regional	11	4,3
		Não foram regulados por centrais de regulação	10	3,9
		Municipal	7	2,7
		Não sei informar	3	1,2
Dificuldade para internar pacientes	Eixo Assistência em Rede	Teve dificuldades com ambos	175	70,3
		Teve dificuldades com cirurgias e casos não covid-19	32	12,9
		Não teve dificuldade	23	9,2
		Teve dificuldades com internação dos casos covid-19 em enfermaria ou UTI	19	7,6
Regulação de leitos covid - dificuldades no acesso à leitos	Eixo Gestão	Insuficiência de leitos de enfermaria, suporte ventilatório ou UTI	137	53 ,7
		Insuficiência de leitos associados a outros fatores	60	23,5
		Não foram enfrentadas dificuldades no acesso aos leitos covid-19	38	14,9
		Não sei informar	11	4,3
		Outros problemas que não a insuficiência de leitos	9	3,5
Situações enfrentada junto ao setor privado	Eixo Gestão	Situações diversas enfrentadas com rede privada – leitos e outros recursos	107	42,0
		Nenhuma	77	30,2
		Situações enfrentada com a rede privada não envolvendo leitos de internação	56	22,0
		Situações enfrentada com a rede privada exclusivamente envolvendo leitos de internação	15	5,9
Regulação de leitos covid - protocolos utilizados	Eixo Gestão	Mais de um protocolo	109	42,7
		Protocolo estadual	91	35,7
		Protocolo regional	28	11,0
		Protocolo municipal	19	7,5
		Não sei responder	4	1,6
		Não utilizou protocolos	2	0,8
		Protocolo nacional	2	0,8
Criação de novos serviços na rede assistencial	Eixo Assistência em Rede	Sim	212	83,1
		Não	39	15,2
Estratégias adotadas nos serviços de urgência e emergência	Eixo Assistência em Rede	Áreas/fluxos específicos para atendimento de covid nos serviços já existentes	179	70,2
		Criados ou adaptados serviços para atendimento covid	131	51,3
		Destinados serviços de urgência específicos para atendimento covid	43	16,8
		Município não dispõe de serviço de urgência	29	11,3
		Não houve mudança na rede de urgência	25	9,8
		Foram destinadas unidades do Samu específicas para atendimento covid	17	6,6
		Implantado teleatendimento para os serviços de urgência	14	5,4
		Não sei informar	3	1,1

Cross: Central de Regulação da Oferta e Serviços de Saúde do Estado de São Paulo; UTI: unidade de terapia intensiva; Samu: Serviço de Atendimento Móvel de Urgência.

**Tabela 3:** Regulação ambulatorial / não covid e outros fatores.

			n	%
Estratégias de regulação adotadas	Eixo Gestão	Múltiplas estratégias	139	54,5
		Outras estratégias de regulação ambulatorial	39	15,3
		Qualificação / monitoramento de filas de espera,	28	11,0
		Não foi realizada nenhuma ação,	18	7,1
		Implantação de protocolos de classificação de risco	14	5,5
		Não sei informar	12	4,7
		Telerregulação	3	1,2
		Matriciamento	2	0,8
Alteração na oferta de consultas especializadas	Eixo Assistência em Rede	Diminuiu	187	73,3
		Aumentou	36	14,1
		Manteve-se estável	29	11,4
		Não sei informar	3	1,2
Alteração na oferta de exames de apoio diagnóstico	Eixo Assistência em Rede	Diminuiu	163	63,9
		Aumentou	58	22,7
		Manteve-se estável	31	12,2
		Não sei informar	3	1,2
Alteração na oferta de procedimento terapêuticos	Eixo Assistência em Rede	Diminuiu	172	67,5
		Aumentou	47	18,4
		Manteve-se estável	28	11,0
		Não sei informar	8	3,1
Alteração na oferta de cirurgias	Eixo Assistência em Rede	Diminuiu	210	82,4
		Aumentou	31	12,2
		Não sei informar	8	3,1
		Manteve-se estável	6	2,4
Alteração na oferta de teleatendimento	Eixo Assistência em Rede	Aumentou	93	36,5
		Não sei informar	72	28,2
		Diminuiu	54	21,2
		Manteve-se estável	36	14,1
Recursos financeiros foram suficientes?	Eixo Gestão	Sim	138	54,1
		Não	100	39,2
		Não sei informar	17	6,7
Alterações na força de trabalho - pronto atendimento e Samu	Eixo Gestão	Houve aumento de pessoal	116	45,5
		Não houve alteração	80	31,4
		Não sei responder	30	11,8
		Houve diminuição de pessoal, incluindo afastamentos e recusas	29	11,4
Alterações na força de trabalho - hospitalar	Eixo Gestão	Houve aumento de pessoal	125	49,0
		Não houve alteração	57	22,4
		Não sei responder	47	18,4
		Houve diminuição de pessoal, incluindo afastamentos e recusas	26	10,2
Alterações na força de trabalho - atenção especializada	Eixo Gestão	Não houve alteração	134	52,5
		Houve diminuição de pessoal, incluindo afastamentos e recusas	45	17,6
		Houve aumento de pessoal	40	15,7
		Não sei responder	34	13,3
		Houve diminuição de pessoal	2	0,8
Dificuldades na aquisição de materiais/ insumos	Eixo Gestão	Múltiplas dificuldades	220	86,3
		Falta de produtos no mercado	14	5,5
		Não houve dificuldades	8	3,1
		Preços abusivos	6	2,4
		Entraves administrativos / jurídicos para aquisição	4	1,6
		Insuficiência financeira / orçamentária municipal	2	0,8
		Não sei responder	1	0,4

Samu: Serviço de Atendimento Móvel de Urgência.

**Tabela 4:** Associação com porte.

Variáveis		Porte				Valor de cramer	p-valor
		Extra grande	Grande	Médio	Pequeno		
Recursos financeiros foram suficientes?	Não	4	25^a^	44^a^	27^a^	0,309	< 0,001
	Não sei informar	0	2	8	7		
	Sim	2	8^a^	31^a^	97^a^		
Regulação de leitos covid feita através de central de regulação?	Estadual/Cross	1^a^	15	52	80	0,315	< 0,001
	Municipal	3^a^	4^a^	0	0^a^		
	Não foram regulados por centrais de regulação	0	1	3	6		
	Não sei informar	0	0	0	3		
	Regional	0	0	4	7		
	Regulados por mais de uma central	2	15	24	35		
Regulação de leitos covid - protocolos utilizados	Mais de um protocolo	1	20	34	54	0,312	< 0,001
	Não sei responder	0	1	0	3		
	Não utilizou protocolos	0	0	1	1		
	Protocolo estadual	0	7^a^	36	48		
	Protocolo municipal	5^a^	6^a^	5	3^a^		
	Protocolo nacional	0	0	1	1		
	Protocolo regional	0	1	6	21^a^		
Regulação de leitos covid - dificuldades no acesso à leitos	Insuficiência de leitos associados a outros fatores	0	12	26^a^	22^a^	0,241	< 0,001
	Insuficiência de leitos de enfermaria, suporte ventilatório ou UTI	1	14	48	74		
	Não foram enfrentadas dificuldades no acesso aos leitos covid-19	3^a^	2	6^a^	27^a^		
	Não sei informar	1	2	3	5		
	Outros problemas que não a insuficiência de leitos	1	5^a^	0^a^	3		
Regulação ambulatorial - acesso não-covid	Implantação de protocolos de classificação de risco	2^a^	1	7	4	0,191	0,146
	Matriciamento	0	0	1	1		
	Múltiplas estratégias	4	25^a^	42	68		
	Não foi realizada nenhuma ação.	0	1	2^a^	15^a^		
	Não sei informar	0	1	5	6		
	Outras estratégias de regulação ambulatorial	0	3	13	23		
	Qualificação / monitoramento de filas de espera	0	4	12	12		
	Telerregulação	0	0	1	2		
Situações enfrentada junto ao setor privado	Nenhuma	0	1^a^	26	50^a^	0,224	< 0,001
	Situações enfrentada com a rede privada não envolvendo leitos de internação	0	6	12^a^	38^a^		
	Situações enfrentada com a rede privada - envolvendo leitos de internação	1	3	5	6		
	Situações enfrentadas com rede privada envolvendo leitos entre outros	5^a^	25^a^	40	37^a^		
Dificuldade para internar pacientes	Não sei informar	1	1	1	3	0,180	0,017
	Não teve dificuldade	0	2	3^a^	18^a^		
	Teve dificuldades com ambos	3	24	69^a^	79^a^		
	Teve dificuldades com cirurgias e casos não covid-19	2	6	7	17		
	Teve dificuldades com internação dos casos covid-19 em enfermaria ou UTI	0	2	3	14^a^		
Alterações na força de trabalho - pronto atendimento e Samu	Houve aumento de pessoal	5	22^a^	45^a^	44^a^	0,195	0,001
	Houve diminuição de pessoal, incluindo afastamentos e recusas	0	7	10	12		
	Não houve alteração	1	4^a^	23	52^a^		
	Não sei responder	0	2	5^a^	23^a^		
Alterações na força de trabalho - hospitalar	Houve aumento de pessoal	5	25^a^	53^a^	42^a^	0,259	0,000
	Houve diminuição de pessoal, incluindo afastamentos e recusas	0	6	10	10		
	Não houve alteração	0	1^a^	7^a^	49^a^		
	Não sei responder	1	3	13	30^a^		
Alterações na força de trabalho - atenção especializada	Houve aumento de pessoal	1	3	20^a^	16	0,194	0,004
	Houve diminuição de pessoal	0	1	1	0		
	Houve diminuição de pessoal, incluindo afastamentos e recusas	1	13^a^	15	16^a^		
	Não houve alteração	3	17	41	73		
	Não sei responder	1	1	6	26^a^		
Dificuldades na aquisição de materiais/ insumos	Entraves administrativos/jurídicos para aquisição	0	0	2	2	0,203	0,026
	Falta de produtos no mercado	1	0	3	10		
	Insuficiência financeira/orçamentária municipal	0	1	0	1		
	múltiplas dificuldades	4	33	78^a^	105^a^		
	Não houve dificuldades	0	1^a^	0^a^	7^a^		
	preços abusivos	0	0	0	6		
Alteração na oferta de consultas especializadas	Aumentou	0	4	15	17	0,076	0,882
	Diminuiu	5	26	59	97		
	Manteve-se estável	1	4	9	15		
	Não sei informar	0	1	0	2		
Alteração na oferta de exames de apoio diagnóstico	Aumentou	1	5	27^a^	25	0,171	0,008
	Diminuiu	4	25	49	85		
	Manteve-se estável	0	5	7	19		
	Não sei informar	1^a^	0	0	2		
Alteração na oferta de procedimento terapêuticos	Aumentou	1	4	19	23	0,073	0,903
	Diminuiu	5	25	53	89		
	Manteve-se estável	0	4	9	15		
	Não sei informar	0	2	2	4		
Alteração na oferta de cirurgias	Aumentou	1	3	13	14	0,108	0,443
	Diminuiu	4	30	68	108		
	Manteve-se estável	0	1	2	3		
	Não sei informar	1	1	0	6		
Alteração na oferta de teleatendimento	Aumentou	5^a^	18^a^	28	42	0,140	0,094
	Diminuiu	0	4	16	34		
	Manteve-se estável	1	5	10	20		
	Não sei informar	0	8	29	35		

Samu: Serviço de Atendimento Móvel de Urgência; UTI: unidade de terapia intensiva.

^a^ Dados estatisticamente significativo mediante análise dos resíduos padronizados ajustados.

### Regulação Hospitalar/Urgência

A maioria dos municípios (58,0%) regulou leitos exclusivos para covid-19 por meio da Central de Regulação da Oferta e Serviços de Saúde do Estado de São Paulo (Cross). Contudo, houve dificuldades no acesso: 53,7% relataram insuficiência de leitos e 70,3% enfrentaram problemas para internar pacientes. Quanto aos protocolos de internação, 42,7% usaram mais de um protocolo, conforme demonstrado na Tabela 2.

A criação de novos serviços para casos suspeitos de covid-19, do tipo centros de atendimento para enfrentamento da covid-19, foi relatada por 83,1% dos respondentes. Em serviços de urgência, 70,2% implantaram fluxos específicos e 51,3% criaram ou adaptaram serviços para atendimento covid-19. A relação entre os setores público e privado foi ampliada, incluindo a expansão do número de leitos (42,0%). Em contrapartida, 21,5% dos gestores públicos receberam pedidos de transferência da rede privada para leitos públicos.

### Regulação Ambulatorial/Não covid

No acesso à saúde ambulatorial não relacionada à covid-19, 54,5% dos respondentes utilizaram múltiplas estratégias de regulação, como monitoramento de filas, protocolos de classificação de risco, matriciamento e telerregulação. Houve redução em consultas especializadas, procedimentos terapêuticos, exames diagnósticos e cirurgias, conforme demonstrado na Tabela 3.

### Outros Fatores Associados à Regulação

Outros fatores interferiram no acesso à saúde durante a pandemia, como a disponibilidade de recursos financeiros, considerada suficiente por 54,0% dos municípios, enquanto 39,0% discordaram. Houve aumento de pessoal no pronto atendimento e Serviço de Atendimento Móvel de Urgência (Samu) (45,5%) e no setor hospitalar (49,0%), mas na atenção especializada 52,5% relataram nenhuma alteração e 17,6% registraram redução devido a afastamentos. Além disso, 86,3% enfrentaram dificuldades na aquisição de materiais e insumos.

### Associações com o Porte dos Municípios

A Tabela 4 apresenta a associação entre o porte do município e as variáveis de interesse. Em relação aos leitos hospitalares, a maioria dos municípios de pequeno porte foi regulada por uma central estadual/Cross (p < 0,001). Além disso, significância estatística foi identificada com a utilização de protocolos para regulação de leitos covid-19 (p < 0,001). Municípios de pequeno porte foram mais propensos a adotar múltiplos protocolos, enquanto municípios de grande porte com maior frequência adotaram o protocolo estadual.

Quanto às estratégias para garantir o acesso a serviços ambulatoriais não relacionados à covid-19, houve associação significativa entre municípios de grande porte e o uso de múltiplas estratégias (p < 0,05), como regulação ambulatorial, qualificação de filas, protocolos de risco, matriciamento e telerregulação. Municípios de pequeno porte, por outro lado, apresentaram associação com a ausência de ações (p < 0,05). Pequenos e médios municípios também adotaram várias estratégias, mas sem significância estatística (p = 0,146).

Nas variáveis de oferta de serviços ambulatoriais, como consultas especializadas e cirurgias, as associações foram menos evidentes. Houve significância estatística na redução de exames diagnósticos (p = 0,008), mas não em consultas especializadas. A suficiência de recursos financeiros foi associada ao porte do município (p < 0,001), com pequenos municípios relatando maior suficiência. Já gestores de grandes e médios municípios relataram insuficiência.

Houve também associação significativa entre porte municipal e aumento de pessoal em serviços de urgência e Samu (p = 0,001) e na atenção hospitalar (p = 0,000). Pequenos municípios tiveram mais “inalteração” da força de trabalho e enfrentaram mais dificuldades na aquisição de insumos, especialmente devido à falta de produtos no mercado (p = 0,026).

Os resultados apresentados foram categorizados entre barreiras e facilitadores do acesso, destacando os arranjos tecnológicos de regulação utilizados, auxiliando na análise da importância desses mecanismos. Nesta etapa do estudo, foram destacadas narrativas dos gestores que contribuem para a compreensão da dinâmica do uso de arranjos na facilitação e enfrentamento das barreiras encontradas na emergência sanitária (Figura).

**Figura:** Barreiras e facilitadores da regulação durante a pandemia.

Nota: A identificação das citações utiliza as referências fornecidas pelo software AtlasTi no formato D:C ¶ P D= número do documento, C= número do código e P= número do paragrafo. UTI: unidade de terapia intensiva; Cross: Central de Regulação da Oferta e Serviços de Saúde do Estado de São Paulo.

## DISCUSSÃO

Em uma perspectiva ampliada de acesso à saúde, partindo dos elementos que integram o processo de regulação, os achados descritos neste estudo reforçam barreiras como insuficiência de leitos, dificuldades na internação de pacientes e redução da oferta de serviços ambulatoriais, em consonância com a literatura^23,24^ atual sobre o enfretamento da pandemia. No entanto, facilitadores como a relação entre o setor público e privado e a articulação regional foram revelados. Em relação à distribuição de recursos, a assimetria identificada provocou efeitos distintos em diferentes regiões do Estado.

A pandemia de covid-19 evidenciou a importância dos recursos financeiros na gestão e organização dos sistemas de saúde. No Brasil, o financiamento do SUS é realizado de forma tripartite, porém a distribuição desigual criou desafios significativos^25^. É importante destacar a ocorrência de eleições municipais no Brasil durante o primeiro ano da pandemia, trazendo ainda outros elementos para o cenário político e da alocação de recursos^26^. Os municípios de maior porte populacional, com maiores responsabilidades na gestão da crise, enfrentaram a insuficiência de recursos para ampliar estruturas hospitalares e adensar tecnologicamente suas unidades de saúde. Em contrapartida, os municípios de pequeno porte, que constituem a maioria, relataram que os recursos foram suficientes, embora apresentassem baixa densidade tecnológica. A complexidade e a burocracia também impuseram dificuldades na utilização dos recursos, agravando os problemas no enfrentamento da pandemia.

A gravidade do quadro infeccioso causado pelo vírus SARS-Cov2 indica a centralidade dos leitos hospitalares na construção da resposta à emergência. Segundo o relatório da Secretaria Extraordinária de Enfrentamento à Covid do Ministério da Saúde, 14% dos pacientes com covid-19 evoluíram para quadros graves no Brasil^27^, requerendo recursos de oxigenoterapia e unidades de tratamento intensivo. Frente a isso, houve expansão de 23,5% do número de leitos de unidade de terapia intensiva (UTI) no Brasil nos primeiros seis meses da pandemia^28^. No entanto, ainda que os dados surpreendam, essa ampliação manteve o perfil da distribuição desigual de leitos, em termos regionais e na divisão entre leitos públicos e privados, acentuando a falha estrutural na distribuição de leitos de UTI no Brasil^29^.

O estado de São Paulo, que concentra uma das maiores ofertas de leitos de UTI do país^30^, se tornou o epicentro da pandemia, especialmente sua capital^31^, sendo o primeiro estado brasileiro a ser atingido. As desigualdades em relação à distribuição de leitos em SP estão fortemente vinculadas à discrepância entre o número de leitos públicos e privados, se considerada a população dependente. A insuficiência de leitos foi uma das principais barreiras identificadas e mobilizou o emprego de arranjos de regulação, na tentativa de minimizar os impactos causados.

A organização da resposta por meio da ampliação de leitos está retratada nos dados coletados, onde é possível também identificar os arranjos que foram utilizados para possibilitar a expansão da rede assistencial, atuando como facilitadores do acesso. Nos municípios de grande e extragrande porte, a ampliação de leitos se deu em grande parte por meio da ampliação da estrutura existente, instalação de hospitais de campanha^32^ e contratualização com hospitais privados.

Os municípios de maior porte, se por um lado se beneficiaram pelo fato de já possuírem uma rede hospitalar e de urgência constituída, por outro lado honraram as pactuações regionais, servindo de referência para os municípios de pequeno porte com rede hospitalar e de urgência insuficiente ou até inexistente^33^.

Já os municípios de médio porte utilizaram arranjos como a modificação do perfil assistencial de determinadas alas, ou até do hospital como um todo, como forma de ampliação do acesso a leitos. Nos municípios de pequeno porte, outros arranjos foram implantados para garantir atendimento inicial de urgência, até a transferência para centros maiores, respeitando a lógica regional de assistência e as pactuações realizadas nas diferentes regiões de saúde^33^. Neste contexto, os gestores de municípios de pequeno porte destacaram medidas adotadas que visavam minimizar as dificuldades quanto à internação, criando estruturas provisórias ou de transição, até que fosse possível o acesso ao recurso adequado.

A organização da assistência no estado de São Paulo reforça a importância da articulação regional existente entre os municípios na composição de uma rede de cuidados integrais de base regional, historicamente estabelecida por intermédio de pactuações entre os gestores^11^. No contexto da emergência, a aposta na constituição de regiões e redes adotada em São Paulo serviu como um facilitador para a organização de grades de referência regional, contemplando diferentes níveis de complexidade, desafios à regulação regional, distribuição de recursos e transporte de pacientes.

Como forma de operacionalizar os fluxos/grades estabelecidos para a assistência, emergem as centrais de regulação como engrenagem central na organização da resposta. O predomínio da central de regulação estadual e a adoção de múltiplos protocolos na regulação de acesso a leitos corroboram com a noção de que as CIRs se constituem como espaços potentes de elaboração de novos arranjos assistenciais para superar dificuldades de acesso^34^ e fortalecem a aposta na constituição de regiões e redes como forma de avançar na construção de um sistema de saúde que se debruce sobre os problemas de acesso e adequação da oferta^11^.

Por meio do protagonismo municipal foram colocadas em prática pactuações construídas nos territórios, fruto de esforços de planejamento e gestão compartilhada^14^, forjados no cenário de intensa mobilização política, tensionado por interesses privados, meios de comunicação e poder judiciário. A Cross atuou como instrumento de governança regional na regulação de leitos, servindo de elo entre entes, como tentativa de minimizar o histórico enfraquecimento do seu papel nas esferas de pactuação^13^, fruto do alargamento da participação de prestadores de serviço em espaços de gestão, do distanciamento dos recursos financeiros e do reduzido poder de negociação das regiões. Este cenário foi agravado pela ausência do governo federal na coordenação da resposta à pandemia, relegando a responsabilidade na construção da resposta aos entes subnacionais.

Por outro lado, a rede privada detinha, somente no estado de São Paulo, mais de 50% dos leitos de UTI existentes, conforme dados anteriores à pandemia^29^. O cenário da emergência sanitária intensificou relações já existentes entre o setor público e privado e serviu de oportunidade para o estabelecimento de novos formatos de cooperação. Há um destaque entre os municípios de médio porte que, ao estabelecerem arranjos tecnológicos de regulação para garantir o acesso dos pacientes a leitos especializados, contaram fortemente com o apoio do setor privado, incluindo os hospitais filantrópicos, estabelecendo parcerias ou ampliando acordo existentes para a disponibilização de leitos covid-19.

Entretanto, a rede privada também recorreu à oferta disponibilizada pelo SUS, evidência do colapso também enfrentando pelos hospitais particulares, planos e seguros de saúde^29,35^. Embora tenha sido discutida no Brasil a implantação da fila única para acesso a leitos covid, unificando a oferta pública e privada, bem como a possibilidade de decretos de requisição de leitos pelo poder público, a proposição não avançou^35^.

A ampliação da força de trabalho, crucial para a ampliação do acesso, também representou um grande desafio para os gestores, tanto pela escassez de recursos humanos disponíveis, quanto pela inexperiência e necessidade de capacitação do contingente de trabalhadores em curto espaço de tempo. O alto índice de contaminação dos profissionais, especialmente aqueles identificados como “linha de frente”, limitou as ações, afetando a oferta e consequentemente o acesso aos serviços de saúde^36^. A proteção dos profissionais em vista do risco aumentado de infecção exigiu esforços no provimento de equipamentos de proteção individual e treinamento adequado para o manejo de equipamentos de proteção individual e pacientes^37^.

Além disso, a dificuldade para aquisição de insumos também figura como importante entrave enfrentado, associado à dificuldade de acesso a leitos. Embora estratégias para lidar com a questão de suprimentos tenham sido identificadas, as limitações no suprimento adequado de hospitais promoveram relatos de verdadeiro pesadelo encarado pelos gestores hospitalares, motivados principalmente pela escassez dos produtos, mas também pela prática de preços exorbitantes, que restringiu ainda mais o acesso^38^.

A alteração da oferta de serviços na atenção especializada também foi apontada como desafio. A redução de cirurgias eletivas e consultas especializadas foram apontadas como solução^36,41^ para aumentar a oferta de atendimento, reduzir exposição de usuários e trabalhadores e otimizar recursos, porém acarretou problemas pela diminuição da oferta, dificultando ainda mais o acesso a serviços de saúde historicamente sensíveis e problemáticos^42^. A preocupação com doenças crônicas considera o impacto da covid-19 e a necessidade de retomar o vínculo com pacientes e o tratamento ambulatorial. A redução da oferta ambulatorial produziu efeitos desastrosos na assistência à saúde especializada, ampliando o desafio permanente neste nível de atenção.

A incorporação de mecanismos de saúde digital como parte da estratégia de ampliação do acesso fortaleceu e inovou pela criação de estratégias de telemonitoramento, atendimento multiprofissional, cuidados em saúde mental e manejo clínico de pacientes internados. Importantes ferramentas para educação em saúde foram utilizadas intensivamente, como recursos de vídeo, reuniões por meio de plataformas digitais entre gestores, utilização de aplicativo de mensagem (WhatsApp)^43^ para auxiliar no processo de regulação de leitos, entre outros, ainda que já viessem sendo utilizadas e reconhecidas em outros contextos.

Os dados deste estudo revelam a complexidade e os desafios enfrentados pelo SUS durante a pandemia de covid-19. A insuficiência de leitos e a desigualdade na sua distribuição, evidenciando inequidades entre os setores público e privado, foram problemas centrais que dificultaram a resposta à emergência. Os municípios lideraram esforços para ampliar a oferta assistencial, expandindo e readequando a rede de atenção. Arranjos tecnológicos de regulação do acesso à saúde, como protocolos de acesso e alteração no perfil assistencial, foram cruciais para enfrentar problemas complexos. A articulação regional e o uso de centrais de regulação foram fundamentais para mitigar as dificuldades, reforçando a importância de uma governança compartilhada e bem estruturada.

No entanto, os esforços para ampliar o acesso aos leitos sacrificaram as ações ambulatoriais, reduzindo a oferta e a força de trabalho ambulatorial, concentrando recursos na atenção hospitalar. Arranjos tecnológicos, como matriciamento, qualificação de filas e uso de ferramentas digitais e telemedicina, contribuíram para garantir o acesso. Estudos sobre o acesso à saúde pós-pandemia serão essenciais para entender o impacto desses arranjos e orientar futuras respostas a emergências sanitárias.

O uso de questionário eletrônico é uma limitação do estudo, podendo ter excluído participantes com menor familiaridade digital e dificultado a captação de realidades complexas.

O fortalecimento de espaços de pactuação regional, que operem por mecanismos robustos de governança, pode ser importante para respostas futuras, assegurando mecanismos de assistência em rede e financiamento de ações. A ampliação da força de trabalho e o adequado suprimento de materiais essenciais, também podem contribuir, minimizando os efeitos sobre as ações de saúde ambulatorial especializada.
